# Outcomes of an oral motor and swallowing rehabilitation program in patients with congenital myopathies

**DOI:** 10.1590/2317-1782/e20240294en

**Published:** 2025-07-07

**Authors:** Ana Luísa Capitelli Dornellas, Fernanda Chiarion Sassi, Ana Paula Ritto, Gisele Chagas de Medeiros, Claudia Regina Furquim de Andrade

**Affiliations:** 1 Divisão de Fonoaudiologia, Hospital das Clínicas, Faculdade de Medicina, Universidade de São Paulo – USP - São Paulo (SP), Brasil.; 2 Departamento de Fisioterapia, Fonoaudiologia e Terapia Ocupacional, Faculdade de Medicina, Universidade de São Paulo – USP - São Paulo (SP), Brasil.

**Keywords:** Dysphagia, Rehabilitation, Congenital Myopathy, Muscular Dystrophy, Muscle Strength

## Abstract

**Purpose:**

To identify factors related to swallowing and oral motor skills in patients with congenital myopathies and evaluate the outcomes of an oral motor and swallowing intervention for this population.

**Methods:**

Participants of this study were twenty-six individuals with diagnosed myopathies or muscular dystrophy, referred to the Division of Speech-Language Pathology. Data collection occurred at three time points: pre-treatment, post-treatment, and three-month follow-up. Oral motor and swallowing assessments were performed using validated protocols. Participants completed a speech therapy program for oropharyngeal dysphagia, consisting of four weekly 30-minute sessions, with daily practice encouraged. Each session was supervised by a speech-language pathologist until independent practice was possible.

**Results:**

The study found that patients with congenital myopathies had significant impairments in posture, mobility, oral functions, and feeding, especially in mastication and swallowing of solid foods. The therapy program resulted in substantial improvements at all evaluation points. Significant differences were observed between pre- and post-treatment, and between pre-treatment and the three-month follow-up, as measured by the ASHA NOMS scale. Oral motor skill assessments showed improved scores on all measures of the AMIOFE-E protocol, except mastication.

**Conclusion:**

The rehabilitation program effectively improved oral motor and swallowing functions in patients with congenital myopathies, reducing the risk of pulmonary aspiration and related complications. The therapy program demonstrated to be highly effective for this patient group.

## INTRODUCTION

Neuromuscular diseases comprise a diverse and heterogeneous group of conditions that can affect motor neurons (both upper and lower), peripheral nerves, the neuromuscular junction, and muscles^(1)^. Despite the wide variety of diseases, the term “neuromuscular” is typically used for hereditary or acquired conditions that manifest with motor dysfunction, with slow or rapid progression^(1)^.

Among neuromuscular diseases, this study will focus on the examination of congenital myopathies and dystrophies. Both conditions result in multisystem involvement over time, leading to impairments, disabilities, and high healthcare costs for patients and their families^(2)^. Generally, dystrophies are gradually progressive and are associated with increased muscle degradation with age, whereas myopathies exhibit non-progressive or slowly progressive characteristics^(3)^.

The literature has pointed a prevalence of 1.62 per 100,000 live births for congenital myopathies^(4)^ and of 16.14 per 100,000 individuals for combined muscle dystrophies, based on studies from Europe, Asia, and North America^(5)^. The pathogenesis is determined through genetic studies, with approaches varying according to the observed phenotype. These may include targeted gene tests (single-gene testing, multigene panels) and comprehensive genomic tests (exome sequencing/array, genome sequencing)^(5)^. Laboratory tests, such as creatine kinase, may or may not be altered during the diagnostic workup, and muscle biopsy can be employed, revealing histological patterns such as fiber size variation, degeneration/regeneration, fibrosis, and muscle tissue loss^(6)^. Differentiation is primarily achieved through muscle biopsy, where dystrophic patterns are indicative of muscular dystrophies^(6)^. However, access to diagnostic tests that allow for differential diagnosis is often limited due to high costs^(5)^.

Congenital myopathies (CMs) constitute a heterogeneous group of rare hereditary muscle diseases, typically presenting from birth or early childhood with hypotonia, muscle weakness, skeletal deformities, and sometimes cramps^(7)^. Feeding difficulties and respiratory insufficiency are common; additional features may include microcephaly, ocular anomalies, joint laxity, muscle atrophy, or hypertrophy^(5)^. They are secondary to genetic or metabolic disorders and generally exhibit a slowly progressive or non-progressive course^(3)^. Although the term “congenital” implies that these diseases present symptoms at birth, it has become clear, particularly with advancements in genetic technologies, that there are also late-onset forms of CMs, which should be considered in the differential diagnosis of adult patients with myopathies^(5)^.

In contrast, muscular dystrophies (MD) correspond to a subgroup of myopathies, classified as dystrophic myopathies. These conditions have a broad phenotype, are progressive in nature, and are associated with increased muscle damage with age^(7)^. MD manifest shortly after birth or in childhood and is characterized by hypotonia, delayed motor development, and progressive muscle weakness. As pointed, the clinical and phenotypic presentation is variable and may affect other muscle groups, including the eyelids, cardiac and respiratory muscles, as well as the muscles involved in oral motor processes such as chewing and swallowing^(8)^. A range of genes can cause dystrophy, thus it also encompasses a broad spectrum of phenotypes, with variable involvement of cardiac/respiratory muscles, the central nervous system, and ocular structures^(9)^.

One of the predominant symptoms in neuromuscular disorders is muscle weakness, which can affect one or more muscle groups^(10)^. This symptom can lead to the development of muscle contractures, scoliosis, gait abnormalities, impaired fine motor movements of the hands, dysphagia, dysarthria, malnutrition, and respiratory insufficiency^(10)^. Muscle weakness, due to the broad phenotypic spectrum, can manifest in varying degrees of severity.

Dysphagia in neuromuscular disorders is prevalent, ranging from 40-80%, and can vary significantly based on genetic mutations, prognosis, symptoms, and age^(11)^. Although studies specifically on congenital myopathies are limited, the early identification of swallowing difficulties is often neglected, with attention and referrals typically occurring only when the condition is already moderate to severe^(12)^. Effective early detection and intervention by speech therapy can improve patient outcomes by raising awareness of the muscular aspects of swallowing, reducing the risk of pulmonary complications and nutritional issues through the use of compensatory techniques and oral motor exercises.

Dysphagia in these disorders affects both the oral and pharyngeal phases, which are crucial for transporting food from the mouth to the stomach, and can impact quality of life, social interactions, and increase morbidity and mortality^(13)^. The literature points that dysphagia in chronic muscle disease results from muscle weakness, and that hypotonia of the tongue, lips, and mandibular disorders or structural alterations in the oral cavity can impair the preparation of a cohesive food bolus and even the process of clearing residual bolus particles^(14)^. Hypotonia in other muscle groups involved in safe swallowing, such as the soft palate, suprahyoid muscles, and laryngeal muscles, can lead to functional impairment in swallowing and airway protection^(14)^.

A common finding in neuromuscular disorders is muscle fatigue, resulting from dysfunction in the lower motor unit or sensory nerves, which leads to difficulties in maintaining muscle contraction strength over time^(15)^. As a result, individuals with muscle weakness need to exert more effort to perform the same activities as healthy individuals, leading to reduced endurance and premature fatigue. Current studies support the hypothesis that congenital myopathies also affect the articulatory organs. However, knowledge about the impact of oral motor impairment on the health of individuals with neuromuscular disorders is limited. The focus on dysphagia and myofunctional alterations is pioneering, particularly as a growing number of individuals with neuromuscular diseases are surviving longer due to ongoing advancements in pharmacological and genetic therapies^(10)^.

Thus, given the above, the aim of this study is to characterize factors associated with swallowing and oral motor skills in patients with congenital myopathies and to assess the outcomes of an oral motor and swallowing intervention for this population.

## METHODS

We conducted a prospective, observational longitudinal study approved by the Research Ethics Committee of Hospital das Clínicas, School of Medicine, University of São Paulo (HCFMUSP), Brazil (CAPPesq Process no. 6.079.863). Data collection procedures began only after the Informed Consent was signed by the participants or their respective guardians.

### Participants

The participants were selected using a convenience sampling method, including all consecutive patients who met the eligibility criteria during the data collection period. The study sample consisted of children and adults of both genders diagnosed with congenital myopathies and dystrophies. Diagnosis was confirmed by the medical team of the Division of Neurology at the same hospital, involving laboratory tests, electromyography (EMG), whole exome sequencing, or muscle biopsy. Patients with myopathies were referred to the Division of Oral Myology HCFMUSP, when they presented with swallowing complaints. Referrals for outpatient speech therapy evaluation and treatment occurred from February 2023 to February 2024.

The inclusion criteria for this study were: a confirmed medical diagnosis of myopathy or muscular dystrophy, presence of oropharyngeal dysphagia diagnosed according to validated speech therapy protocols, regardless of severity; use or non-use of alternative feeding methods; and clinical stability according to medical criteria (control of the underlying disease and stable vital parameters appropriate for the respective age group).

The exclusion criteria were: individuals with cognitive impairments or levels of consciousness that hindered comprehension of verbal information required for speech therapy assessment, according to the Mini-Mental State Examination (MMSE)^(16)^; patients residing in other states, making it impossible to attend therapy sessions in person; use of tracheostomy or T-tube; esophageal dysphagia; and head and neck cancer.

Participants who completed all stages of oral motor and swallowing evaluations and passed the cognitive screening were included in the sample. Initially, 59 patients were selected for the study, but only 46 patients were enrolled for the initial assessments based on our inclusion/exclusion criteria (i.e., among the 13 excluded patients, 4 had cognitive impairment and did not pass the MMSE, and 9 had other neurological conditions rather than myopathy or muscular dystrophy). Of the 46 patients initially enrolled, 26 completed the first assessment, 24 completed all four therapy sessions and were assessed for post-treatment results (2 patients were excluded due to non-compliance with the therapy plan), and 20 attended the follow-up evaluation.

### Procedures

All patients included in the study underwent the same assessment procedures at three distinct time points: pre-treatment, post-treatment, and three months after treatment completion (follow-up). The assessment protocols used in all three evaluations are described below.

#### Cognitive screening

In order to be included in the research, the MMSE^(16)^ was administered as a cognitive screening tool. The MMSE is a brief test for individuals aged 15 and older, comprising items on temporal orientation (five points), spatial orientation (five points), registration of three words (three points), attention and calculation (five points), recall of the three words (three points), language (eight points), and visual construction ability (one point). Scores on the MMSE can range from zero (indicating severe cognitive impairment) to a maximum of 30 points (indicating optimal cognitive function). Individuals who presented altered MMSE scores were excluded from the study.

#### Swallowing assessment - Dysphagia Risk Evaluation Protocol (DREP)

The clinical assessment of swallowing was conducted using the Dysphagia Risk Evaluation Protocol (DREP). The DREP^(17)^ is a Brazilian protocol validated and designed to assess the risk of dysphagia by administering controlled volumes (3ml, 5ml, and 10ml) and free volumes (50ml) of water and homogeneous pasty food, with three repetitions each. Results are recorded as “pass” or “fail”, and administration is stopped if the patient shows clinical signs suggesting laryngotracheal penetration or aspiration. The DREP screening version, adopted in the present study, demonstrated excellent validity with sensitivity at 92.9%, specificity at 75.0%, negative predictive values at 95.5%, and an accuracy of 80.9%^(17)^. In the screening version of the DREP the following variables are considered: multiple swallows, altered cervical auscultation, post-swallowing vocal quality changes, coughing, and choking. Individuals who presented any of these signs were considered at risk for bronchial aspiration.

#### Functional level of swallowing

To assess the functional level of swallowing, we used the Functional Oral Intake Scale from the American Speech-Language-Hearing Association National Outcome Measurement System (ASHA NOMS)^(18)^. This multidimensional scale assigns a number from 1 to 7 based on the supervision needed for feeding and the diet level: **Level 1** - Unable to safely swallow orally; receives all nutrition and hydration through alternative feeding methods; **Level 2** - Unable to safely swallow orally for nutrition and hydration; may ingest some consistency in therapy with maximum cues; alternative feeding method required; **Level 3** - Ingests less than 50% of nutrition orally; safe swallowing with moderate cue use or maximum diet restrictions; alternative feeding method necessary; **Level 4** - Safe swallowing with moderate cue use; moderate diet restrictions; alternative feeding method or oral supplement still required; **Level 5** - Safe swallowing with minimal diet restrictions; minimal cue use occasionally; self-monitors during meals; **Level 6** - Independent eating and drinking; minimal cue use; self-monitors when needed; specific food items may need avoidance; extended feeding time possible; **Level 7** - Independent feeding; safe and efficient swallowing across all consistencies; effective use of compensatory strategies.

The ASHA NOMS scale categorizes individuals based on their ability to safely swallow and manage diets, aiding in treatment planning and monitoring of dysphagia interventions.

#### Oral motor clinical assessment

The Expanded Protocol of Orofacial Myofunctional Evaluation with Scores (OMES-E) was used for the clinical oral motor assessment^(19)^. This protocol, based on prior evaluation models, incorporates numerical scales to reflect the physical characteristics and orofacial behaviors of the subjects. It evaluates components of the stomatognathic system (lips, tongue, mandible, and cheeks) in terms of posture and position (maximum score: 64), mobility (maximum score: 114), and performance during swallowing and mastication (maximum score: 52). The total possible score is 230 points, indicating optimal performance. Data collection was conducted through visual inspection during the assessment and by analyzing records from photos and videos, using a tablet (Samsung Galaxy Tab A - model SM-T295).

In order to evaluate data reliability, all participants were assessed by two experienced speech-language pathologists. The speech-language pathologists assigning the scores of the OMES-E had successfully passed specific training tests. The Kappa Coefficient was used to verify agreement between examiners for the overall scores. The obtained result indicated a high level of agreement (>0.832).

#### Mandibular range of movement

The technique used to measure mandible range of movement was based on the methodology already published in the literature^(20)^. With the use of a digital caliper (Digimess Pró-Fono Digital Caliper), the following measurements (in millimeters) were performed:

1. maximal incisor distance - we measured the distance between the incisive faces of the mandibular and maxillary central incisors;
2. right and left lateral excursion - we measured the horizontal distance between the mandibular central incisor to the maxillary central incisor after asking the individual to glide his/her mandible to the right and then to the left. When there was a midline deviation (i.e. between mandibular and maxillary central incisors), we used the pertaining adjustment;
3. protrusion – for this measurement the patient was asked to glide the mandible forward. We then measured the horizontal overlap value between the mandibular central incisors over the maxillary central incisors.

#### Oral motor and dysphagia rehabilitation

All participants underwent the Speech Therapy Program for Oropharyngeal Dysphagia^(21)^. The selection of techniques used in this research was based on theoretical and scientific foundations, with consideration of the techniques' feasibility according to the patients' profiles. The program aimed to: a) provide instruction and clarification regarding swallowing alterations and associated risks; b) reorganize the motor function of the orofacial muscles (i.e. motor exercises for strength, range of motion, and tongue movement; range of motion and movement exercises for the lips; tongue coordination and mastication exercises; tongue base exercises; prolonged production of the vowel /i/; glottic adduction exercises; blowing through a straw; oral cavity residue removal exercises); c) stimulate sensory functions (i.e. gustatory stimulation; thermo-tactile-gustatory stimulation); d) guide protective maneuvers and facilitating postures for swallowing (i.e. Masako maneuver; chin tuck postural maneuver; effortful swallow); e) modify diets when necessary (i.e. consistency and volume modification).

The program comprised four weekly therapy sessions, each lasting 30 minutes. Daily practices were recommended, and each therapy session was supervised by the treating speech-language pathologist (SLP) until the patient could perform the practices independently. The prescribed regimen included 10 repetitions of each active exercise, to be performed at least once a day, and instructions for using protective maneuvers or modifying postures were to be applied during mealtimes. After each session, patients received pictorial and written instructions outlining the home exercises. Weekly, the treating SLP monitored adherence by observing the patients perform the exercises and reviewing their feedback. Patients also received feedback on their progress and performance during the treatment sessions. Any observed improvements were recorded in the patient's clinical file.

During the three-month unassisted period, patients were encouraged to maintain their daily exercise routines independently. They were provided with clear guidelines to ensure consistent practice and adherence to the prescribed regimen. Patients were also advised to keep a log of their daily exercises and any difficulties encountered, which would be reviewed during follow-up assessments. The data collected from these logs, along with the patients' self-reported adherence and any changes in symptoms, were recorded in their clinical files and used to evaluate long-term treatment outcomes.

### Data analysis

The collected data were subjected to statistical analysis using SPSS software, version 29. Quantitative data underwent descriptive analysis (mean, standard deviation, median, minimum, and maximum for quantitative variables, and total count and percentage for qualitative variables) and inferential analysis to compare the results obtained at each of the three assessment time points—pre-treatment, immediately post-treatment, and at the three-month follow-up (Friedman test for paired samples/repeated measures ANOVA, with post-hoc pairwise analysis and Bonferroni correction if significant). A significance level of 5% was adopted for all analyses.

## RESULTS

The characterization of the patient sample in relation to clinical and demographic variables is described in Table 1, which provides a descriptive summary of the demographic data and baseline clinical data of the 26 participants in the sample. The mean age at the time of the oral motor and swallowing pre-treatment assessments was 42.5 years, with ages ranging from 12 to 72 years, and a higher prevalence of participants over 18 years of age and female patients. The study included an equal number of participants diagnosed with either myopathies or congenital muscular dystrophies. Among the participants with congenital myopathy, one was diagnosed with nemaline myopathy, one with inflammatory myopathy, and one with myopathy associated with a tropomyosin 3 gene mutation. Among the participants with muscular dystrophy, four were diagnosed with Steinert’s disease, two with facioscapulohumeral dystrophy, three with Emery-Dreifuss muscular dystrophy, three with limb-girdle muscular dystrophy, and one with muscular dystrophy due to a GGPS1 mutation.

**Table 1:** Demographic and baseline clinical data (n=26)

**Demographic / clinical variable**	**Descriptive summary**
Age (years)	
Mean ± SD	42.5 ± 18.5
Mediana (min; max)	48.0 (12.0; 72.0)
Gender, n (%)	
Female	18 (69.2%)
Male	8 (30.8%)
Diagnosis, n (%)	
Dystrophy	13 (50.0%)
Congenital Myopathy	13 (50.0%)

Caption: n = number of participants; SD = standard deviation; % = percentage

Table 2 presents the comparison of swallowing assessment results obtained at three time points: pre-treatment, post-treatment, and follow-up, as measured by the DREP. Signs and symptoms not observed in any patients were excluded from the analysis. No significant differences were detected across these time points (p>0.05 according to the Friedman test for paired samples), with the exception of cough.

**Table 2:** Comparison of results according to the Dysphagia Risk Evaluation Protocol (DREP) at pre-treatment, post-treatment, and follow-up

**Variables**	**Results**		
	Pre-treatment (n=26)	Post-treatment (n=24)	Follow-up (n=20)
Multiple swallows, n (%)	8 (29.6%)	4 (14.8%)	2 (7.4%)
Overall comparison	**p=0.097**		
Cough, n (%)	6 (22.2%)	0 (0.0%)	0 (0.0%)
Overall comparison	***p=0.018*** *		
Choke, n (%)	4 (12.1%)	0 (0.0%)	0 (0.0%)
Overall comparison	**p=0.368**		

* Significant comparison between measures according to the Friedman test for paired samples (repeated measures ANOVA)

Caption: n = number of participants; % = percentage

Table 3 displays the results of the comparison for the functional level of swallowing according to the Functional Oral Intake Scale from the American Speech-Language-Hearing Association's National Outcome Measurement System (ASHA NOMS), obtained at pre-treatment, post-treatment, and at the three-month follow-up. Significant differences were observed between the measures at each time point (p < 0.05 according to the Friedman test for paired samples), with post-hoc pairwise analysis and Bonferroni correction revealing that these differences were significant between the pre-treatment and post-treatment measures (p<0.05) and between the pre-treatment and three-month follow-up measures (p<0.05). No significant differences were found in the post-hoc pairwise analysis between the post-treatment results and the follow-up results after three months (p>0.05).

**Table 3:** Comparison of the results according to the functional level of swallowing at pre-treatment, post-treatment, and follow-up

**ASHA NOMS Levels**	**Results**		
	Pre-treatment (n=26)	Post-treatment (n=24)	Follow-up (n=20)
Level 4, n (%)	1 (3.7%)	0 (0.0%)	0 (0.0%)
Level 5, n (%)	12 (44.4%)	4 (14.8%)	4 (14.8%)
Level 6, n (%)	12 (44.4%)	13 (48.1%)	6 (22.2%)
Level 7, n (%)	1 (3.7%)	7 (25.9%)	10 (37.0%)
Overall comparison	***p<0.001*** *		
**Pairwise** comparison	pre-treatment vs. post-treatment: p=0.022**		
	pre-treatment vs. follow-up: p=0.002**		
	post-treatment vs. follow-up: p=0.385		

* Significant comparison between measures according to the Friedman test for paired samples (repeated measures ANOVA);

** Significant comparison between measures according to post-hoc pairwise analysis with Bonferroni correction

Caption: ASHA NOMS = American Speech-Language-Hearing Association’s National Outcome Measurement System; n = number of participants; % = percentage

It is important to note that the ASHA NOMS swallowing scale measures the level of supervision and adaptation required for feeding and the diet level. Throughout the proposed rehabilitation program, no participants included in the final analysis used an alternative feeding method; all nutrition and hydration were administered orally. However, the use of cues and adaptations were necessary.

Table 4 shows the comparison of results obtained pre-treatment, post- treatment, and at follow-up using the Orofacial Myofunctional Evaluation Protocol with Scores (AMIOFE-E). Significant differences were observed in the AMIOFE-E scores at each of these time points for nearly all sections (p<0.05 according to the Friedman test for paired samples), with the exception of the 'Functions: Chewing' section. Table 4 also displays the results of the post-hoc pairwise analysis with Bonferroni correction for the AMIOFE-E protocol. No statistically significant differences were found between the scores obtained at the post-treatment re-evaluation and those obtained at the three-month follow-up for any section of the AMIOFE-E protocol.

**Table 4:** Comparison of the results according to the AMIOFE-E at pre-treatment, post-treatment, and follow-up

**Variables**	**Results**		
	Pre-treatment (n=26)	Post-treatment (n=24)	Follow-up (n=20)
**Posture and Position**			
Mean ± SD	53.7 ± 5.1	55.7 ± 5.8	55.8 ± 5.7
Median (min; max)	54.5 (42.0; 64.0)	57.0 (43.0; 64.0)	56.0 (47.0; 63.0)
Overall comparison	p=0.013*		
Pairwise comparison	pre-treatment vs. post-treatment: p=0.052**		
	pre-treatment vs. follow-up: p=0.007**		
	post-treatment vs. follow-up: p=0.465		
**Mobility**			
Mean ± SD	78.7 ± 17.0	94.9 ± 14.9	91.5 ± 19.0
Mean (min; max)	77.5 (48.0; 109.0)	97.0 (68.0; 114.0)	96.0 (56.0; 114.0)
Overall comparison	***p=0.002*** *		
Pairwise comparison	pre-treatment vs. post-treatment: p<0.001**		
	pre-treatment vs. follow-up: p=0.015**		
	post-treatment vs. follow-up: p=0.330		
**Performance swallowing**			
Mean ± SD	22.3 ± 2.9	24.2 ± 2.7	23.6 ± 2.5
Mean (min; max)	23.0 (18.0; 27.0)	24.0 (20.0; 28.0)	24.0 (19.0; 28.0)
Overall comparison	p=0.009*		
Pairwise comparison	pre-treatment vs. post-treatment: p=0.004**		
	pre-treatment vs. follow-up: p=0.089		
	post-treatment vs. follow -up: p=0.224		
**Performance mastication**			
Mean ± SD	15.4 ± 2.8	18.3 ± 4.9	16.2 ± 3.0
Mean (min; max)	15.5 (11.0; 20.0)	17.5 (13.0; 39.0)	16.0 (8.0; 20.0)
Overall comparison	**p=0.062**		
**Overall score for swallowing/mastication**			
Mean ± SD	40.6 ± 6.0	46.1 ± 5.6	43.5 ± 4.8
Mean (min; max)	41.5 (21.0; 49.0)	45.0 (37.0; 63.0)	44.0 (31.0; 52.0)
Overall comparison	p<0.001*		
Pairwise comparison	pre-treatment vs. post-treatment: p<0.001**		
	pre-treatment vs. follow-up: p=0.015**		
	post-treatment vs. follow-up: p=0.144		
**Total score AMIOFE-E**			
Mean ± SD	173.1 ± 21.7	196.7 ± 21.0	190.8 ± 22.2
Mean (min; max)	172.5 (138.0; 219.0)	196.5 (156.0; 230.0)	197.0 (153.0; 222.0)
Overall comparison	p<0.001*		
Pairwise comparison	pre-treatment vs. post-treatment: p<0.001**		
	pre-treatment vs. follow-up: p=0.006**		
	post-treatment vs. follow-up: p=0.123		

* Significant comparison between measures according to the Friedman test for paired samples (repeated measures ANOVA);

** Significant comparison between measures according to post-hoc pairwise analysis with Bonferroni correction

Caption: AMIOFE-E = Orofacial Myofunctional Evaluation Protocol with Scores; n = number of participants; SD = standard deviation; min = minimum; max = maximum

Table 5 displays the comparison of mandibular range of motion measurements obtained at three assessment points: pre-treatment, post-treatment, and at follow-up. No significant differences were found between the measurements at these time points (p>0.05 according to the Friedman test for paired samples), except for the measure of left mandibular lateralization. The finding that there were no significant differences between the measurements at the various time points, except for the measure of left mandibular lateralization, suggests that this result may be a single, isolated occurrence rather than a meaningful or clinically relevant pattern. Given that this is the only significant finding among the multiple comparisons, it might lack robustness and could be attributed to random variation rather than reflecting a true effect. In the context of the broader analysis, this isolated result should be interpreted with caution, as it may not indicate a substantial or generalizable difference in mandibular range of motion across the treatment period. Table 5 also includes the results of the post-hoc pairwise analysis with Bonferroni correction for these measurements.

**Table 5:** Comparison of mandibular range of motion measurements at pre-treatment, post-treatment, and follow-up

**Measurements**	**Results (mm)**		
	Pre-treatment (n=26)	Post-treatment (n=24)	Follow-up (n=20)
**Maximal incisor distance**			
Mean ± SD	40.9 ± 10.2	42.1 ± 9.5	42.2 ± 7.3
Mean (min; max)	40.6 (19.8; 61.5)	42.8 (28.3; 63.0)	46.2 (32.8; 60.8)
Overall comparison	**p=0.523**		
**Right excursion**			
Mean ± SD	8.0 ± 4.0	8.3 ± 3.5	9.9 ± 5.7
Mean (min; max)	7.2 (1.3; 12.6)	7.8 (1.1; 15.4)	6.8 (3.1; 23.2)
Overall comparison	**p=0.822**		
**Left excursion**			
Mean ± SD	7.7 ± 4.4	9.2 ± 5.1	8.9 ± 5.5
Mean (min; max)	7.0 (0.6; 16.5)	7.5 (1.8; 20.3)	7.7 (1.1; 20.7)
Overall comparison	***p=0.024*** *		
Pairwise comparison	pre-treatment vs. post-treatment: p=0.759		
	pre-treatment vs. follow-up: p=0.014**		
	post-treatment vs. follow-up: p=0.032**		
**Protrusion**			
Mean ± SD	5.9 ± 3.2	6.7 ± 4.1	5.8 ± 2.5
Mean (min; max)	5.3 (1.7; 13.0)	4.8 (0.3; 15.2)	6.8 (2.4; 10.9)
Overall comparison	**p=0.974**		

* Significant comparison between measures according to the Friedman test for paired samples (repeated measures ANOVA);

** Significant comparison between measures according to post-hoc pairwise analysis with Bonferroni correction

Caption: n = number of participants; mm = millimeters; SD = standard deviation; min = minimum; max = maximum

## DISCUSSION

Overall, the results of our study revealed that individuals diagnosed with congenital myopathies experience significant impairments in posture, mobility, oromyofunctional organ function, and feeding performance, particularly in the mastication and swallowing of solid consistencies. The proposed therapeutic program led to substantial improvements across the three evaluation points: pre-treatment, post-treatment, and a three-month follow-up. Distinct differences were observed in the comparisons between pre- and post-treatment, as well as between pre-treatment and the three-month follow-up, based on the ASHA NOMS scale. Furthermore, the assessment of oral motor skills showed higher scores in all measures of the AMIOFE-E protocol at each time point, except when considering mastication.

The analysis of clinical and demographic data revealed that the majority of patients included in this study were female. A 2023 review study^(22)^ identified that neuromuscular diseases may differ between men and women, with certain neuromuscular disorders tending to manifest earlier and with more severe muscle atrophy in men, as observed in myotonic dystrophy type I. This was also found in the study by Dogan et al.^(23)^, where a slight majority of the total sample of 1,409 patients with myotonic dystrophy type I (53.15%) were women; however, men tended to exhibit more pronounced symptoms, such as myotonia, cognitive impairment, and cardiac and respiratory complications, while women experienced more extramuscular issues, such as cataracts, obesity, thyroid dysfunction, and gastrointestinal symptoms. Other neuromuscular disorders are more frequently observed in women, which may be explained by genetic and hormonal factors. For instance, a study involving patients with myotonic dystrophy type 2^(24)^ found that proximal weakness was more severe and more common in women, possibly due to decreased hormone levels after menopause, a key period for the decline of muscle function in women.

Regarding age, most individuals included in this study received a late diagnosis. Studies suggest that some of the conditions examined in this work, despite being hereditary, may have late-onset manifestations^(25)^. This can lead to delays in achieving an accurate diagnosis, which subsequently postpones referral to multidisciplinary rehabilitation. Such delays may explain the average age of the patients in this study, directly affecting the rehabilitation process and the quality of life of individuals with congenital myopathies and dystrophies^(24)^.

In this study, the Dysphagia Risk Evaluation Protocol (DREP)^(17)^ identified the most recurrent signs of impaired swallowing of thin liquid consistencies, specifically multiple swallows, coughing, and choking. These symptoms are likely linked to muscle weakness in the tongue and buccinator muscles, leading to insufficient bolus propulsion, ineffective motor control within the oral cavity, and respiratory incoordination, which disrupts the timing required for swallowing apnea^(10)^. The DREP^(17)^ analysis revealed that, while no significant differences were observed across the three evaluation points (pre-treatment, post-treatment, and three-month follow-up), the therapy was particularly effective in addressing the sign of coughing. This suggests that the effects of speech therapy were long-lasting, as patients no longer exhibited coughing, while some residual symptoms, such as multiple swallows and choking, persisted at a lesser degree.

In terms of clinical swallowing evaluation, statistically significant improvements were noted when comparing swallowing levels using the ASHA NOMS scale^(18)^, which measures both the level of supervision needed during feeding and the safety of the diet. During the entire speech therapy program, none of the patients required alternative feeding methods; all received nutrition and hydration orally, although some adjustments and cues were necessary throughout the treatment. In comparing pre and post-treatment assessment results and pre-treatment results with those obtained at the three months follow-up, no significant variations were observed. This indicates that patients' performance did not deteriorate during the follow-up period. Additionally, no statistically significant differences were found between post-treatment evaluations and those conducted three months later. This suggests that the therapy retains its effectiveness over the intermediate term, with no loss of the improvements achieved during the oral motor and swallowing rehabilitation program. Moreover, the results obtained from the ASHA NOMS scale^(18)^ indicate that the improvements observed in this scale at post-treatment and the three-month follow-up can be attributed to enhancements in the dynamics of swallowing following the therapy.

Existing literature indicates that muscular weakness significantly impacts the orofacial myofunctional system, leading to impairments in mastication and swallowing functions^(26)^. As already pointed, these issues are often observed in patients with congenital myopathies and can be attributed to anatomical and functional abnormalities such as craniofacial disproportions and dentofacial alterations, which hinder precise coordination of masticatory movements and swallowing responses^(27)^. In alignment with these findings, our study utilized the AMIOFE-E protocol^(19)^ to assess these functions. The evaluation revealed significant differences across pre-treatment, post-treatment, and three-month follow-up assessments for nearly all sections, except for mastication The lack of significant change in this section may be due to persistent difficulties in forming a cohesive food bolus, linked to weakness in the masseter and buccinator muscles and imprecise tongue movements^(14)^.

Moreover, it is important to observe that the total score for the AMIOFE-E protocol^(19)^ increased following treatment, highlighting overall improvement. However, the characteristics of mastication and swallowing remained the most affected, potentially due to congenital myopathy-related craniofacial disproportions and dentofacial abnormalities. This aligns with Havner et al.^(28)^ findings, which reported a high prevalence of open lip posture and labial hypotonia in patients with myotonic dystrophy type 1 (DM1), and a lower prevalence in Duchenne muscular dystrophy (DMD). These results underscore the complex interplay between muscle weakness and functional impairments in the orofacial system.

The AMIOFE-E protocol^(19)^, while effective for assessing oral motor skills in the general population, was not specifically designed to address the unique needs of patients with congenital myopathy. As of the conclusion of this study, no dedicated protocol exists for evaluating oral motor functions and swallowing conditions specific to congenital myopathy patients. This gap highlights the need for specialized assessment tools to better address and understand the challenges faced by this particular patient group.

Regarding mandibular range and mobility measurements, this study found no significant differences, which may be attributed to the dentofacial alterations observed in this population. Such alterations have been documented in the literature^(29)^, where craniofacial and dentofacial growth abnormalities are associated with difficulties in masticatory function and an increased duration of oral ingestion. Although not statistically significant, the maximum oral opening measurements were below the normative range reported for healthy individuals (40-60 mm)^(20)^. This observation can be explained by the literature, which notes that individuals with myopathy and muscular dystrophy may exhibit dentofacial disproportion^(26)^.

Alterations in swallowing can significantly impact nutritional status and overall health, often leading to increased morbidity and mortality. However, research on the progression and severity of dysphagia, as well as longitudinal data on its rehabilitation, is sparse^(30)^. This gap in knowledge is crucial for informing discussions with families and multidisciplinary teams about early intervention with alternative feeding methods and strategies to reduce the risk of aspiration and adverse clinical outcomes. In this study, oral motor and swallowing rehabilitation proved effective in improving posture, mobility, and oral function, and in reducing the risk of aspiration according to the DREP protocol^(17)^. Follow-up evaluations conducted three months after the completion of the therapeutic plan showed that participants did not exhibit further improvements, indicating that the gains achieved during therapy were not sustained in the longer term. This may be attributed to several factors that require further investigation, including the fact that the follow-up consultations occurred only after a three-month gap, which may have influenced the continuity of care.

The present study has several limitations. While the use of a convenience sampling method, including all consecutive patients who met the eligibility criteria, ensured feasibility and a consistent approach to participant selection, this method presents inherent limitations. Primarily, convenience sampling may limit the generalizability of the findings to broader populations, as the sample may not fully represent all individuals who could meet the criteria under different circumstances. Additionally, the participants were recruited from a single institution, which means the procedures and outcomes might reflect specific institutional protocols and practices, potentially limiting the applicability of the findings to other settings. Furthermore, the consecutive selection of participants might introduce selection bias, particularly if certain external factors influenced patient availability or eligibility during the data collection period. Despite these limitations, this study is one of the few to characterize factors associated with swallowing and oral motor skills in patients with congenital myopathies and to assess the outcomes of an oral motor and swallowing intervention for individuals with diagnosed myopathies or muscular dystrophy. These findings contribute valuable insights and highlight areas for further investigation to improve care for these populations.

Future research should focus on longitudinal studies to evaluate the sustained effects of therapy, the need for additional interventions, and the potential adaptation of dietary consistency or compensatory maneuvers. Imaging assessments of swallowing should be considered as complementary diagnostic tools for oropharyngeal dysphagia, and including a control group could strengthen the statistical evidence. Furthermore, developing a telehealth follow-up program with monthly consultations could improve therapeutic adherence and management in a more efficient timeframe.

## CONCLUSION

Based on the findings, it can be concluded that the rehabilitation program implemented in this study was effective for managing oral motor and swallowing alterations in this specific group of patients. The application of the rehabilitation program resulted in significant improvements in swallowing and oral motor functions compared to the initial assessment. These results demonstrate that the therapy effectively reduces the risk of pulmonary aspiration and associated clinical complications, underscoring its efficacy for this patient population.
